# Molecular Cloning and Expression Profile of a Halloween Gene Encoding Cyp307A1 From the Seabuckthorn Carpenterworm, *Holcocerus hippophaecolus*

**DOI:** 10.1673/031.013.5601

**Published:** 2013-06-20

**Authors:** Jiao Zhou, Haolin Zhang, Juan Li, Xia Sheng, Shixiang Zong, Youqing Luo, Kentaro Nagaoka, Qiang Weng, Gen Watanabe, Kazuyoshi Taya

**Affiliations:** 1 The Key Laboratory for Silviculture and Conservation, Ministry of Education, College of Forestry, Beijing Forestry University, Beijing 100083, P.R. China.; 2 Laboratory of Animal Physiology, College of Biological Science and Technology, Beijing Forestry University, Beijing 100083, PR China.; 3 Faculty of Agriculture, Tokyo University of Agriculture and Technology.

**Keywords:** ecdysteroid, prothoracic gland, Q-RT-PCR, spook

## Abstract

20-Hydroxyecdyone, an active form of ecdysteroid, is the key hormone in insect growth and development. Halloween genes encode ecdysteroidogenic enzymes, including cytochrome P450 monooxygenase. CYP307A1 (spook) is accepted as an enzyme acting in the so-called ‘black box’ that includes a series of hypothetical and unproven reactions that finally result in the oxidation of 7-dehydrocholesterol to diketol. In this study, the *Holcocerus hippophaecolus* Hua (Lepidoptera: Cossidae) CYP307A1 (*HhSpo*) gene was identified and characterized. The obtained cDNA sequence was 2084 base pairs with an open reading frame of 537 animo acids, in which existed conserved motifs of *CYP450* enzymes. The transcript profiles of *HhSpo* were analyzed in various tissues of final instar larvae. The highest expression was observed in the prothoracic gland, while expression level was low but significant in other tissues. These results suggest that the sequence character and expression profile of *HhSpo* were well conserved and provided the basic information for its functional analysis.

## Introduction

Insect molting and metamorphosis are regulated by steroid hormones named ecdysteroids (Gilbert et al. 2002; Spindler et al. 2009), which regulate larval-larval molts and, in holometabolous insects, metamorphic molts to the pupa and adult. These processes are coordinated and controlled by a polyhydroxylated steroid, 20-hydroxyecdysone (20E), the precursor of which is ecdysone (E) (Rewitz et al. 2006c; De Loof 2008). The ecdysteroid biosynthesis concludes with several hydroxylations catalyzed by cytochrome P450 enzymes. The cytochrome P450 enzymes encoded by the Halloween genes (*spook, spo; phantom, phm; disembodied, dib*; *shadow, sad*; *shade, shd*) catalyze a series of hydroxylation steps resulting in the active molting hormone 20E (Marchai et al. 2010). The Halloween genes have been identified and predicted in many insects, and the functions of these genes have been characterized in *Drosophila melanogaster, Bombyx mori*, and *Manduca sexta* (Iga et al. 2010).

The initial step in 20E biosynthesis is the conversion of cholesterol to 7-dehydrocholesterol. Between this 7-dehydrocholesterol and the first upstream compound exhibiting the highly characteristic ecdysteroid structure, diketol, is the so-called ‘black box’. This black box includes a series of hypothetical and unproven reactions, which result in the oxidation of 7-dehydrocholesterol to diketol (Grieneisen et al. 1993; Namiki et al. 2005; Ono et al. 2006). During this process, the genes *spo* (*CYP307A1*) and *spok* (*CYP307A2*) encoding the enzymes catalyzing these hydroxylations were first identified in *D. melanogaster* using a molecular genetic approach (Warren et al. 1995). To date, several paralogs were found in this sub- family (*CYP307*): *spook* (*spo,CYP307A1*), *spookier* (*spok, CYP307A2*) and *spookiest (spot, CYP307B1*), but their biochemical function is not known (Namiki et al. 2005; Ono et al.2006; Rewitz et al. 2007).

The seabuckthorn carpenterworm, *Holcocerus hippophaecolus* Hua (Lepidoptera: Cossidae), is a destructive forest pest of seabuckthorn, *Hippophae rhamnoides* L. (Rosales: Elaeagnaceae), a shrub widely distributed throughout northern and western regions of China that prevents soil erosion and desertification (Marchai et al. 2011). The larvae seriously obstruct water transportation of seabuckthorn by boring into the trunk and roots. *H. hippophaecolus* has one generation every three to four years, and 16 larval stages occupy most of its life history. The larval and pupal stages both last more than 20 days. It is widely distributed throughout its host's range and mostly damages trees more than five years old. Currently, *H. hippophaecolus* infests seabuckthorn plantations totaling 66,500 hectares in area, often at high levels (Tian et al. 1997; Zhou 2002). The damage is so severe and extensive that the seabuckthorn carpenterworm is considered a major threat to the continued existence of seabuckthorn plantations in China (Luo et al. 2003; Fang et al. 2005). Its voraciousness, high reproduction rate, and hidden behavior makes *H. hippophaecolus* a very difficult pest to control efficiently. Larval development, regulated by an important hormone 20E, is thought to be the key stage in pest control. A complete understanding of regulatory process of 20E is imperative for their rational management.

This paper reports on the molecular cloning and expression profile of *H. hippophaecolus* ortholog of one Halloween gene, CYP307A1 (*spo*), which is predicted as an enzyme acting in the black box uncharacterized conversion steps. Based on the identification of the *HhSpo* sequence, relative tissue and stage specific expression levels were analyzed using QRT-PCR. These results provided the basic information for its functional analysis.

**Table 1.**
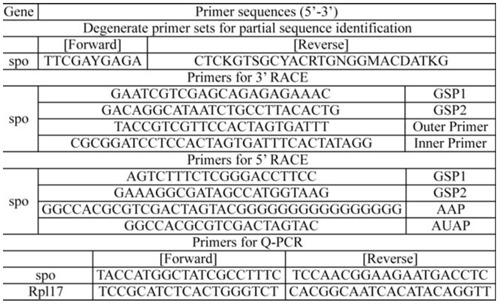
Oligonucleotide primers used for identification of *HhSpo* and quantitative real time PCR.

## Materials and Methods

### Insects

*H. hippophaecolus* from Liaoning province were cultured in a laboratory. The larvae were group-reared on an artificial diet at 26° C under high humidity conditions and a 16:8 L:D cycle (Rybczynski et al. 1994). With this regimen, pupal-adult development took approximately 25 days. Tissues were extirpated under insect saline and rinsed quickly in RNA-later before being flash-frozen and stored at -80° C.

### Total RNA isolation and cDNA synthesis

Tissues were dissected from last instar larvae and adults. Total RNA was extracted using Trizol Reagent (Invitrogen, www.invitrogen.com) according to the protocol. First-strand cDNA was reverse transcribed using 1 *µ*g of total RNA by TIANScript RT Kit (Tiangen, www.tiangen.com).

### Amplification of cDNA fragment

The degenerate primers (Table 1) were designed from highly conserved regions of amino acid sequences from *M. sexta, B. mori,* and *D. melanogaster*. First-strand cDNA from the prothoracic gland was amplified using Taq polymerase (Tiangen). The PCR program included an initial denaturation step of 3 min at 94° C, and then 35 cycles were run as follows: 94° C for 30 sec, 50° C for 30 sec, and 72° C for 1 min, with a final extension of 10 min at 72° C. Amplification products were separated by 1% agarose gel electrophoresis and stained with ethidium bromide, purified using Gel Extraction Mini Kit (Watson Biotechnologies, (Watson, www.walvax.com) Inc. Shanghai). The purified fragment was cloned using pEasy-T1 Cloning Kit (TransGen, www.transgen.com.cn) and Trans 5α Chemically Competent Cell (TransGen). Positive clones were verified by colony PCR, and several of these clones were sequenced.

### Rapid amplification of cDNA ends (3′ RACE and 5′ RACE)

The 3′ RACE was performed using the 3′-Full RACE Core Set Ver. 2.0 (Takara, www.takara-bio.com). Gene specific primers (Table 1) and Taq polymerase (Tiangen) were used for nested PCR under the following conditions: an initial denaturation at 94° C for 3 min, followed by 35 cycles of 94° C for 30 sec, 55° C for 30 sec, and 72° C for 1 min, and a final extension at 72° C for 10 min. The PCR product was excised, sub-cloned, and sequenced as described above.

The 5′ RACE was conducted with BD SMART™ cDNA Amplification Kit (Clontech, www.clontech.com). Gene specific primers (Table 1) and Taq polymerase (Tiangen) were used for nested PCR under the following conditions: an initial denaturation at 94° C for 3 min, followed by 30 cycles of 94° C for 30 sec, 66.5° C for 30 sec, and 72° C for 2 min, with a final extension at 72° C for 10 min. All the gene-specific primers used in 3′RACE and 5′ RACE were designed utilizing Primer Premier 5.0 (www.PremierBiosoft.com).

### Phylogenetic analysis

The amino acid sequences used in the phylogenetic tree come from different organisms and were retrieved from GenBank database. Multiple sequence alignments were performed using Clustal X software (Thompson et al. 1997). A phylogenetic tree was constructed by MEGA version 4.0 (Tamura et al. 2007) using the neighbor-joining method (Saitou and Nei 1987) with a bootstrap test of 1000 replications.

### Quantitative real time PCR analysis of gene expression

Gene expression of *HhSpo* was analyzed by Q-RT-PCR using a real-time light-cycler (BIORAD, www.bio-rad.com). Tissues dissected from three to 10 individuals were pooled from larvae and adults to analyze expression in the following tissues: prothoracic glands, midgut, Malpighian tubules, ganglia, brain, fat body, epidermis, muscle, adult ovary, and testes. The final instar (16^th^ instar larvae) lasts for 20 days, and the transcript level in different days (D4, D6, D8, D12, D14 and D16) of final instar was performed to show the developmental expression in the prothoracic gland. Three to seven independent samples were used, representing each day. Primers for Q-RT-PCR analysis were designed using the Primer 3 program (Rozen et al. 2000) (Table 1). The final PCR reactions contained 0.4 mM of each primer, 1X SsoFast EvaGreen (Invitrogen), and 3 µl DNA template, in a final volume of 10 µl. All quantitative reactions were subjected to 95° C for 30 sec, followed by 35 cycles at 95° C for 5 sec, 55° C for 10 sec, and 72° C for 10 sec. Melting curve analysis was applied to all reactions to ensure homogeneity of the reaction product. In addition, the amplified size was checked by electrophoresis and then sequenced. Transcript levels of the target genes were normalized to the *Manduca* ribosomal gene *rpL17A* after correcting for differences in amplification efficiency.

## Results

### Molecular cloning and phylogenetic analysis of *HhSpo*

**Figure 1.**
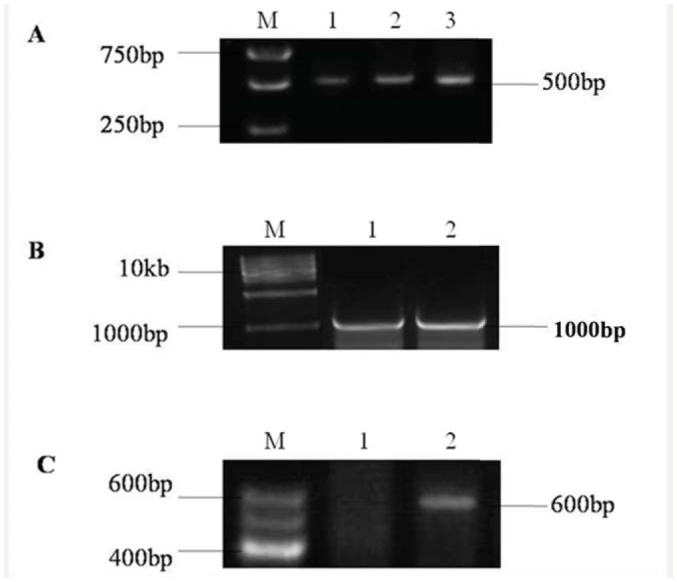
The full-length fragment of *Holcocerus hippophaecolus* CYP307A1 (*Spo*) was obtained by RT-PCR and RACE technology. (A) The gradient PCR amplification of middle fragment of *HhSpo* gene by degenerate primers. Line 1 to Line 3 represents different annealing temperature, 55° C , 58° C , and 61 °C . (B) The 5′RACE result of *HhSpo* gene (1000 bp). Line 1 and Line 2 are the PCR results amplified by GSP2 and AAP. The annealing temperature of Line 1 and Line 2 are 53° C and 55° C. (C) The 3′RACE result of *HhSpo* gene (600 bp). Line 1 is the PCR result amplified by GSP1 and Outer Primer. Line 2 is the PCR result amplified by GSP2 and Inner Primer. High quality figures are available online.

**Figure 2.**
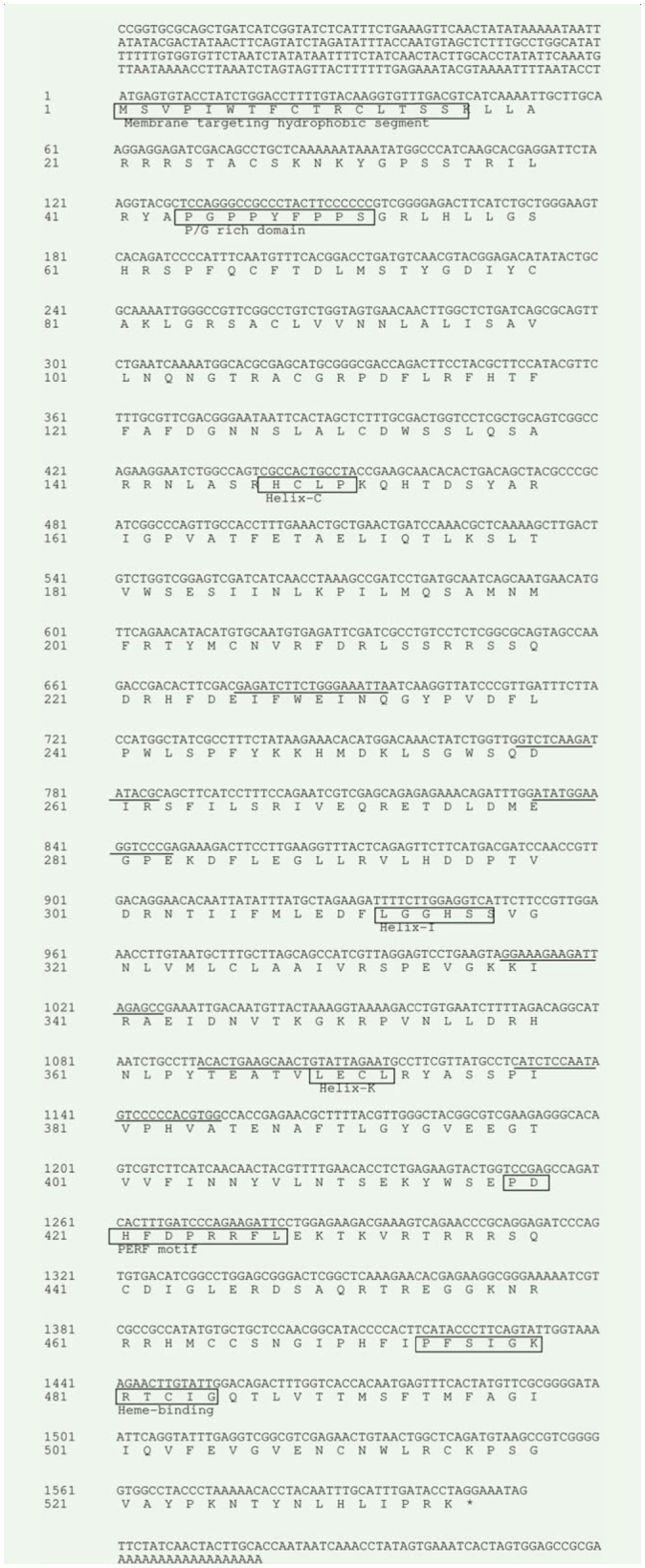
The complete nucleotide and deduced amino acid sequence of the CYP307A1 (*Spo*) of *Holcocerus hippophaecolus*. The start codon ATG is indicated with bold and the stop codon TGA is indicated with bold and by an asterisk. The underlined nucleotides show the positions of gene specific primers used in the experiment. The characteristic P450 structure, P/G rich domain following a membrane targeting hydrophobic segment and the conserved P450 motifs were shown by the boxed amino acids. High quality figures are available.

**Figure 3.**
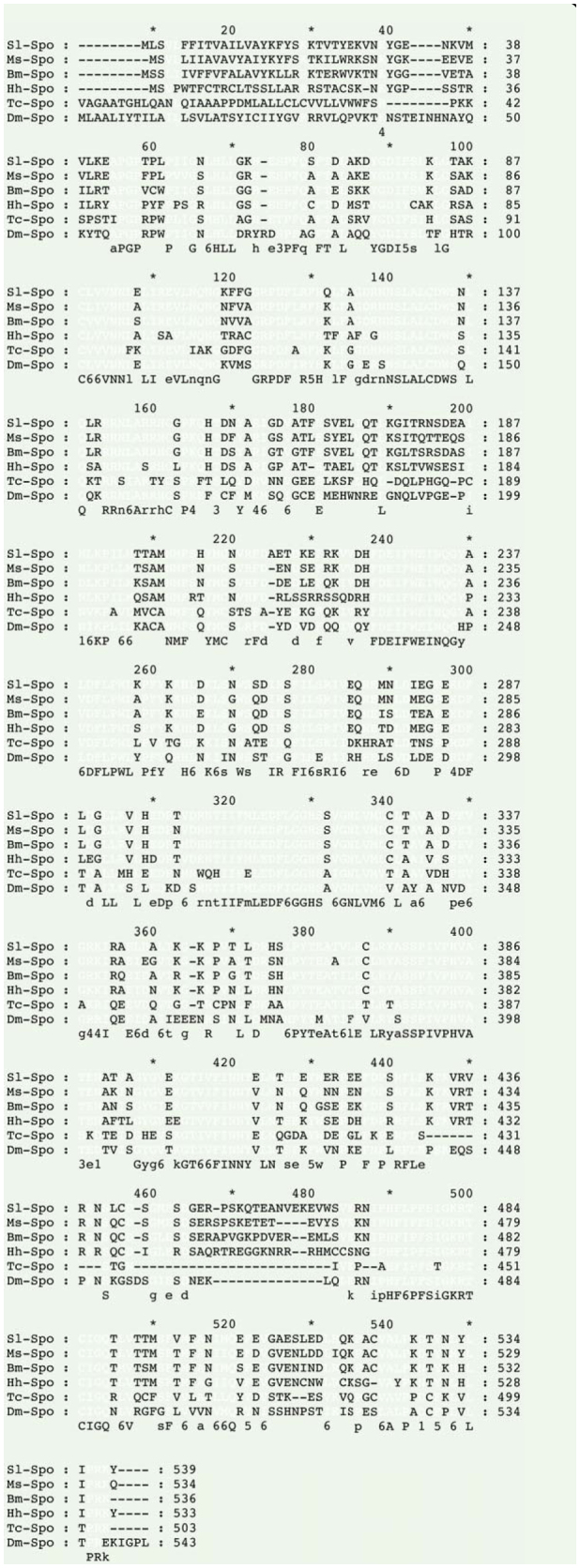
Amino acid sequence alignment of *HhSpo*. Residues in black were identities in the figure. Sl, *Spodoptera littoralis* (Gen Bank accession no. ACY92457.1); Ms, *Manduca sexta* (Gen Bank accession no. AB174778); Bm, *Bombyx mori* (Gen Bank accession no. BAH47267); Hh, *Holcocerus hippophaecolus*; Tc, *Tribolium castaneum* (Gen Bank accession no. EFA1 1558); Dm, *Drosophila melanogaster* (Gen Bank accession no. AAF50766). High quality figures are available.

A primary fragment, approximately 500 bp, of *H. hippophaecolus spo* gene was amplified by RT-PCR using a pair of degenerate primers (Figure 1A). Longer sequences extending into the 5′-UTR and encompassing the 3′-UTR were obtained by RACE using gene specific primers. The full-length cDNA of *HhSpo* was 2084 bp, which contained an open reading frame of 537 amino acids (Figure 1B, C). Analysis showed the deduced protein sequences of *HhSpo* exhibited typical P450 characteristics (Figure 2). WxxxR, of which the arginine is thought to form a charge pair with the propionate of the heme, is located in helix-C. Helix-I (AGxxT) corresponds to a proton transfer groove on the distal side of the heme. ExxR, located in the helix-K, stabilizes the core structure of the enzyme through a set of salt bridge interactions. A fourth conserved motif is the aromatic region, or ‘PERF’ motif (PxxFxPxRF). Finally, the heme-binding loop (PFxxGxRxCxG) includes a conserved cysteine, which serves as ligand to the heme iron. This extremely conserved loop is often considered as the signature for P450 proteins (Feyereisen 1999; Werck et al. 2000; Simonet et al. 2004). In the *HhSpo* sequence, WxxxR motif of helix-C and Helix-I motif (AGxxT) were not well conserved compared with the other three motifs (Figure 3).

Sequence alignment revealed that the length of the coding region of the *HhSpo* gene com-pared with those of homologs from other organisms was highly conserved. However, the *HhSpo* protein had 76%, 54%, 60%, 69%, and 75% identity with the homologs of *B. mori* (BAH47267), *Tribolium* castaneum(EFAl 1558), *D. melanogaster* (AAF50766), *M. sexta* (ABI74778), and *Spodoptera littoralis* (ACY92457), respectively (Figure 3).

**Figure 4.**
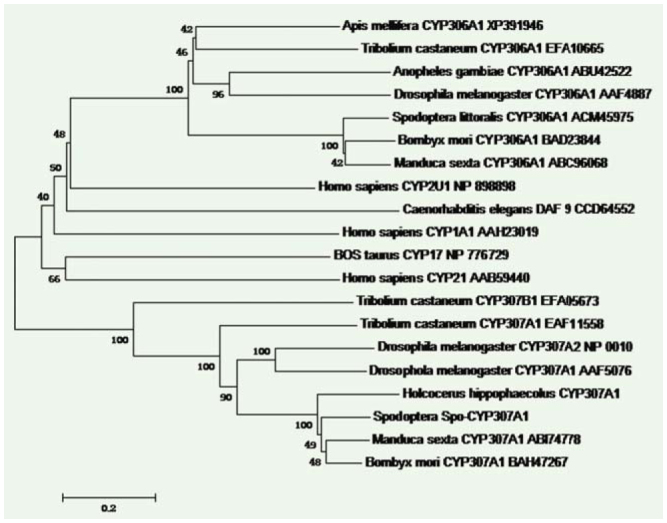
Phylogenetic tree of Halloween gene *Spo*. The tree was generated based on the whole amino acid sequences by neighbor-joining method using Mega program with a bootstrap value of 1000 trials for each branch position, excluding the gap position. The indicated numbers are bootstrap values as a percentage of 1000 replicates, and the scale bar indicates 0.2 change per residue. The bootstrap values more than 50% are indicated. High quality figures are available online.

A phylogenetic tree was constructed using the ORF amino acid sequences of *Spo* in *H. hippophaecolus, T. castaneum, M. sexta, B. mori, D. melanogaster*, and *S. littoralis*, in addition to some other genes of the *CYP2* clan from other insects, vertebrates, and *C. elegans* in order to probe ancestral relationships and the origin of the *CYP2* clan involved in steroid biogenesis. The phylogenetic tree was clearly separated into three clusters of *CYP307* (*Spo*like genes), *CYP306* (*phm*), and another group composed with Human *2U1*, Human *CYP1A1*, Human *CYP21*, Bovine *CYP17*, and *C. elegans DAF9* (Figure 3). The identity and similarity between *HhSpo* and other insects, such as *Drosophila Spo* (Diptera) and *Tribolium Spo* (Coleoptera), were relatively low compared to *Manduca Spo* (Lepidoptera) (Figure 3). The overall amino acid identity of deduced orthologous proteins was made up of insects belonging to three orders (Diptera, Coleoptera, and Lepidoptera) and ranged from an average of 49% for *Spo*-like proteins to a somewhat lower value of 46% for *Phm* proteins (Figure 4).

### Relative tissue- and stage-specific expression profile of *HhSpo*

**Figure 5.**
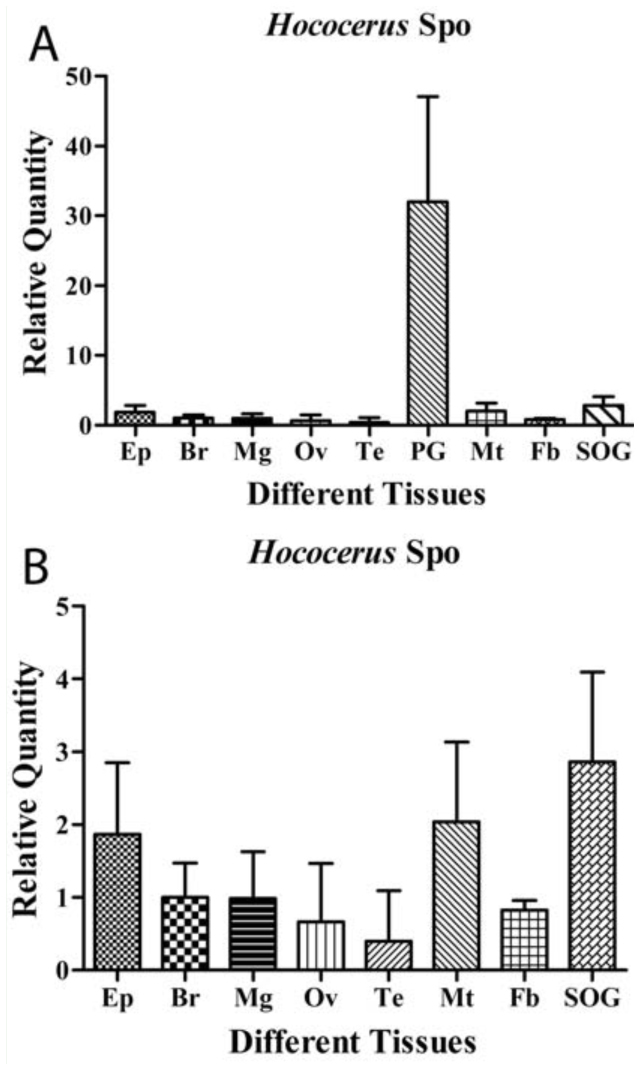
(A) The relative *HhSpo* transcript level measured in different larval tissues and adult gonads using Q-RT-PCR. (B) The *HhSpo* transcript level in various tissues except the prothoracic gland. All larval tissues were dissected from 14-day-old final instar laval, and adult tissue were from two-day-old female ovaries and male testes. The data represented means of three independent pools (three times per pool), which were run in duplicate using Q-RT-PCR and normalized to rpL17A transcript levels. Ep: epidermis; Br: brain; Mg: midgut; Ov: female ovaries; Te: male testes; PG: prothoracic glands; Mt: Malpighian tubules; Fb: fat body; SOG: suboesophageal ganglion. The vertical bars indicated S.E.M. (n = 3–5, measuring 4–6 individual samples per measurement). High quality figures are available online.

Q-RT-PCR was employed to study the tissuespecific (prothoracic gland, midgut, Malpighian tubules, brain, fat body, epidermis, ovaries, and testes) and stage-specific (D4, D6, D8, D12, D14, and D16 of final instar larvae) expression profile of *HhSpo*. Figure 5A shows predominant expression in the prothoracic gland. Likewise, compared with the high transcript level in the prothoracic gland, *HhSpo* exhibited lower levels in the epidermis, brain, Malpighian tubules, midgut, fat body, and suboesophageal ganglion. Trace amounts of transcripts were found in the adult ovaries and testes (Figure 5B).

**Figure 6.**
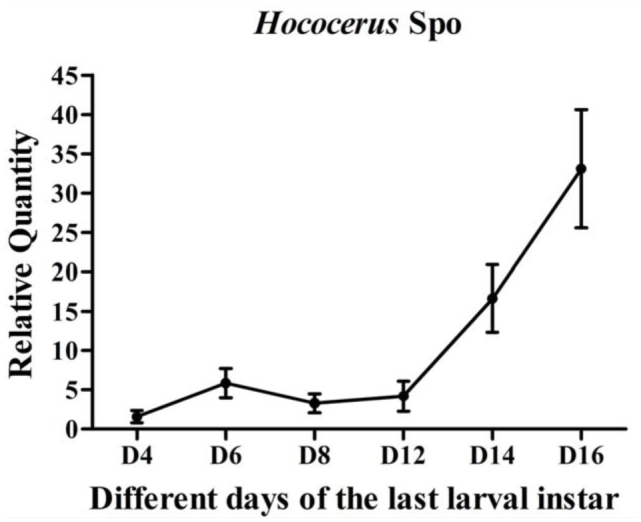
The relative *HhSpo* transcript level measured in the prothoracic gland during the fifth larval development. The data represented means of three independent pools of six animals, which were run in duplicate using Q-RT-PCR and normalized to rpL17A transcript levels. High quality figures are available online.

**Figure 7.**
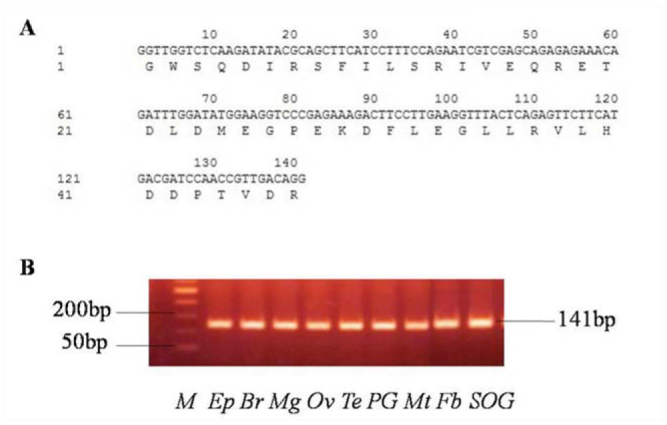
(A) Elelctrophoretic analysis of Q-RT-PCR products in specific tissues. Ep: epidermis; Br: brain; Mg: midgut; Ov: female ovaries; Te: male testes; PG: prothoracic glands; Mt: Malpighian tubules; Fb: fat body; SOG: suboesophageal ganglion. (B) The amino acid and nuclear acid sequence of Q-RT-PCR product of *HhSpo*. High quality figures are available online.

Based on the tissue distribution of *HhSpo* provided in Figure 5, the transcript level throughout final larval development was studied, and the results are given in Figure 6. The relative expression patterns of *HhSpo* in the prothoracic gland started at a low level at the D4, was followed by a small increase until D6, kept stable from D6 to D8, gently decreased until D12, and dramatically increased to its peak at D16. However, the relative mRNA level appeared more flat throughout the formal D6–D12. In order to verify the accuracy of Q-RT-PCR results, the amplified products were checked by electrophoresis and then sequenced. An approximate 140 bp-signal was detected in the prothoracic gland, epidermis, brain, Malpighian tubules, midgut, fat body, sub oesophageal ganglion, ovaries, and testes (Figure 7B). Together with the amino acid and nuclear acid sequence of the Q-RT-PCR product of *HhSpo* (Figure 7A), the Q-RT-PCR results were confirmed to be accurate.

## Discussion

Apart from the recent report made on the Halloween genes in holometabolous insects, this study is the first characterization and report on the relative transcript levels of Halloween orthologs in the forest pest *H. hippophaecolus*. This study identified that *HhSpo* was expressed specifically in the prothoracic gland, and analyzed the transcript profile in specific tissues and stages, which suggested that *CYP307A1 (Spo)* had an essential func-tion in ecdysteroid biosynthesis in *H. hippophaecolus*.

The charaterization of the Halloween gene *spo* emerged from molecular genetic studies of *D. melanogaster*, and then the presence of this gene was confirmed in other insect species (Niwa et al. 2004; Niwa et al. 2005; Rewitz et al. 2007). In this study, the characterization of *HhSpo* was described, while the WxxxR motif of Helix-C, and the Helix-I motif (AGxxT) in all typical P450 motifs were not well conserved, as previously described in other insects (Niwa et al. 2005; Ono et al. 2006; Iga et al. 2010). The identity of the whole sequence of *spo* was different among the species, but their important domains of P450 enzymes were well conserved (Niwa et al. 2005; Rewitz et al. 2006c; Iga et al. 2010). In insects, *Spo* and *Phm* belong to the same *CYP* family, *CYP2* clan (Gilbert et al. 2002). In the phylogenetic analysis of this study, sequences of steroidogenic *CYP2* clan from vertebrates and *C. elegans* were included to probe ancestral relationships and the origin of *CYP2* clan involved in steroid biogenesis. As Figure 3 shows the steroidogenic *CYP2* clan of insects was evolutionarily related to vertebrates and *C. elegans* steroidogenic since they clustered in two major groups of *CYP306A1* and *CYP307* (*Spo*- like gene), those related to Human *2U1*, Human *CYP1A1*, Human *CYP21*, Bovine *CYP17*, and *C. elegans DAF9*. Therefore, it is likely that different steroidogenic *CYP* enzymes were derived from common ancestors and were recruited for steroid biosynthesis prior to the protostome-deuterostome split, which has been show in previous studies (Rewitz et al. 2008; Markov et al. 2009). From the phylogenetic analysis, it can be concluded that *HhSpo* belongs to *CYP2* clan and is well-conserved in both vertebrates and invertebrates.

In contrast to *phm, dib, sad*, and *shd*, for which each insect geonome carried one ortholog, several paralogs of *spo*-like (*CYP307*) genes had been formed by duplications, which were believed to mediate the same enzymatic reaction (Namiki et al. 2005; Ono et al. 2006; Sztal et al. 2007). In the phylogenetic analysis (Figure 4), the overall amino acid identity for *Spo*-like proteins showed higher values compared with *Phm* proteins, which indicated that *Spo*-like proteins were the most highly conserved of *CYP2* clans. The reason that *Spo*-like genes were more conserved than the other arthropod steroidogenic *CYP* enzymes is not known, although it might be related to the possibility that *Spo* acted in the rate-limiting black box reaction(s) (Gilbert et al. 2002; Lafont et al. 2005). Thus, the evolutionary conservation on the Halloween genes shows their importance for normal growth and development in holometabolous insects (Marchai et al. 2010).

In this study, the expression level that *HhSpo* predominantly detected in the prothoracic gland compared with other tissues proved that the prothoracic gland was the main source for ecdysteroid biosynthesis in *H. hippophaecolus*. Previously described *in situ* hybridization and Q-RT-PCR studies in *D. melanogaster, M. sexta, S. Iittoralis*, and *B. mori* found the Halloween genes *Spo* to be mostly expressed in the prothoracic cells of the ring gland and in the prothoracic gland of immature stages (Namiki et al. 2005; Ono et al. 2006; Rewitz et al. 2006a, 2006c, 2007; Iga et al. 2010). However, the prothoracic gland is not the only source for ecdysteroid biosynthesis, as other tissues can perform ecdysteroid synthesis, and ecdysteroids can also play major roles in the reproductive physiology of adult insects (Verlinden et al. 2000; Simonet et al. 2004). In this study, a lower *HhSpo* transcript level was detected in the epidermis, brain, Malpighian tubules, midgut, fat body, and suboesophageal ganglion. This suggests that other tissues may have roles in ecdysteroid biosynthesis. According to previous studies, Malphigian tubules may release 20E into the hemolymph, whereas the midgut accumulates polar ecdysteroid metabolites prior to their excretion (Feyereisen et al. 1978, 1980; Rewitz et al. 2006b), which indicates that the Malpighian tubules may function not only in the excretion of 20E but also in maintaining the hemolymph 20E titer that is elicited during molting to the pupa. Moreover, recent work showed that 20E was involved in the differentiation of stem cells from the midgut of the caterpillar *S. littoralis* (Smagghe et al. 2005). These may imply that in the Lepidoptera, larval-pupal-adult metamorphosis is a complex process and ecdysteroids act on peripheral tissues.

The prothoracic gland undergoes apoptosis and usually disappears before adult stage. Subsequently, in adult insects, ecdysteroid production is taken over by the gonads (ovaries/testes) (Dubrovsky 2005). In this study, trace amounts of *HhSpo* transcripts were also found in the adult ovaries and testes, which was consistent with the importance of 20E for normal oogenesis (Raikhel et al. 2004). Marchai (2010) pointed out that the ovaries were the primary source of ecdysteroids in adult females, where the ecdysteroids influence reproduction and are incorporated as conjugates into the eggs for future embryonic development. In late larval and adult males, the testes also appear to be capable of producing the hormone (Marchai et al. 2010). In the course of this study, only the tissues produced ecdysteroid in adults, although a low *HhSpo* transcript level was determined in the ovaries and testes, which may indicate that ecdysteroids are involved in reproduction. The specific function of ecdysteroids in adults still needs further study, especially in males.

Whether a molt leads to only cuticle shedding or the entire restructuring of the body plan, it is initiated by 20E. The hemolymph ecdysteroid titers in *Drosophila* described that the surges of 20E secretion occurred in midembryogenesis, before each larval molt, before pupariation, and during terminal differentiation of the adult structures (Dubrovsky 2005). In this study, the analysis of *HhSpo* expression during the final larval development was consistent with the 20E titer in *Drosophila*. The first small peak of *HhSpo* around D6 and the second and large peak at D16 were consistent with the surges of 20E in *Drosophila*, representing a metamorphic molting and the molting to the pupa respectively. All developmental transitions, such as larva-to-larva, larva-to-pupa, and pupa-to-adult, were initiated by 20E. At the end of larval development, the 20E signals arrived at a peak (Dubrovsky 2005).

In summary, *HhSpo* was highly conserved in Lepidoptera. The expression patterns suggested importance of *HhSpo* in ecdysone biosynthesis by prothoracic glands and gonads. In order to test whether *CYP307A1* can catalyze cholesterol and cholesterol derivatives (22-hydroxycholesterol and 25-hydroxycholesterol), further experiments need to be done. Moreover, Cyp307a1 is a candidate enzyme for controlling the rate-limitng step of ecdysteroid biosynthesis. Thus, elucidating the biochemical role of *Cyp307A1* will be one of the key research areas in future studies of ecdysteroid biosynthesis.

## Abbreviations

**HhSpo**,: *Holcocerus hippophaecolus* CYP307A1
**Q-RT-PCR**,: quantitive reverse transcriptase polymerase chain reaction
**Spo**,: *spook*
**Spok**,: *spookier*
**Spot**,: *spookiest*
